# Management of bleeding following major trauma: an updated European guideline

**DOI:** 10.1186/cc8943

**Published:** 2010-04-06

**Authors:** Rolf Rossaint, Bertil Bouillon, Vladimir Cerny, Timothy J Coats, Jacques Duranteau, Enrique Fernández-Mondéjar, Beverley J Hunt, Radko Komadina, Giuseppe Nardi, Edmund Neugebauer, Yves Ozier, Louis Riddez, Arthur Schultz, Philip F Stahel, Jean-Louis Vincent, Donat R Spahn

**Affiliations:** 1 Department of Anaesthesiology, University Hospital Aachen, RWTH Aachen University, Pauwelsstrasse 30, 52074 Aachen, Germany; 2 Department of Trauma and Orthopedic Surgery, University of Witten/Herdecke, Hospital Cologne Merheim, Ostmerheimerstrasse 200, 51109 Cologne, Germany; 3 Faculty of Medicine in Hradec Králové, Department of Anaesthesiology and Intensive Care Medicine, University Hospital Hradec Králové, 50005 Hradec Králové, Czech Republic; 4 Accident and Emergency Department, University of Leicester, Infirmary Square, Leicester LE1 5WW, UK; 5 Department of Anaesthesia and Intensive Care, University of Paris XI, Faculté de Médecine Paris-Sud, 63 rue Gabriel Péri, 94276 Le Kremlin-Bicêtre, France; 6 Department of Emergency and Critical Care Medicine, University Hospital Virgen de las Nieves, ctra de Jaén s/n, 18013 Granada, Spain; 7 Guy's & St Thomas' Foundation Trust, Westminster Bridge Road, London, SE1 7EH, UK; 8 Department of Traumatology, General and Teaching Hospital Celje, 3000 Celje, Slovenia; 9 Shock and Trauma Center, S. Camillo Hospital, I-00152 Rome, Italy; 10 Institute for Research in Operative Medicine (IFOM), Ostmerheimerstrasse 200, 51109 Cologne, Germany; 11 Department of Anaesthesia and Intensive Care, Université Paris Descartes, AP-HP Hopital Cochin, Paris, France; 12 Department of Surgery and Trauma, Karolinska University Hospital, 171 76 Solna, Sweden; 13 Ludwig-Boltzmann-Institute for Experimental and Clinical Traumatology and Lorenz Boehler Trauma Center, Donaueschingenstrasse 13, 1200 Vienna, Austria; 14 Department of Orthopaedic Surgery and Department of Neurosurgery, University of Colorado Denver School of Medicine, Denver Health Medical Center, 777 Bannock Street, Denver, CO 80204, USA; 15 Department of Intensive Care, Erasme University Hospital, Université Libre de Bruxelles, Route de Lennik 808, 1070 Brussels, Belgium; 16 Institute of Anesthesiology, University Hospital Zurich, 8091 Zurich, Switzerland

## Abstract

**Introduction:**

Evidence-based recommendations are needed to guide the acute management of the bleeding trauma patient, which when implemented may improve patient outcomes.

**Methods:**

The multidisciplinary Task Force for Advanced Bleeding Care in Trauma was formed in 2005 with the aim of developing a guideline for the management of bleeding following severe injury. This document presents an updated version of the guideline published by the group in 2007. Recommendations were formulated using a nominal group process, the Grading of Recommendations Assessment, Development and Evaluation (GRADE) hierarchy of evidence and based on a systematic review of published literature.

**Results:**

Key changes encompassed in this version of the guideline include new recommendations on coagulation support and monitoring and the appropriate use of local haemostatic measures, tourniquets, calcium and desmopressin in the bleeding trauma patient. The remaining recommendations have been reevaluated and graded based on literature published since the last edition of the guideline. Consideration was also given to changes in clinical practice that have taken place during this time period as a result of both new evidence and changes in the general availability of relevant agents and technologies.

**Conclusions:**

This guideline provides an evidence-based multidisciplinary approach to the management of critically injured bleeding trauma patients.

## Introduction

Uncontrolled post-traumatic bleeding is the leading cause of potentially preventable death among trauma patients [1,2]. About one-third of all trauma patients with bleeding present with a coagulopathy on hospital admission [3-5]. This subset of patients has a significantly increased incidence of multiple organ failure and death compared to patients with similar injury patterns in the absence of a coagulopathy [3,5,6]. Appropriate management of the trauma patient with massive bleeding, defined here as the loss of one blood volume within 24 hours or the loss of 0.5 blood volumes within 3 hours, includes the early identification of potential bleeding sources followed by prompt measures to minimise blood loss, restore tissue perfusion and achieve haemodynamic stability. Confounding factors include co-morbidities, pre-medication and physical parameters that contribute to a coagulopathic state [7,8].

The early acute coagulopathy associated with traumatic injury has recently been recognised as a multifactorial primary condition that results from a combination of shock, tissue injury-related thrombin generation and the activation of anticoagulant and fibrinolytic pathways. The condition is influenced by environmental and therapeutic factors that contribute to acidaemia, hypothermia, dilution, hypoperfusion and haemostasis factor consumption [3,4,8-11]. A number of terms have been proposed to describe the condition, which is distinct from disseminated intravascular coagulation, including acute traumatic coagulopathy [4], early coagulopathy of trauma [5], acute coagulopathy of trauma-shock [8] and trauma-induced coagulopathy [12]. With the evolution of the concept of an early post-traumatic coagulopathic state, it may be appropriate to reassess some data from the past, and with time new research will doubtless lead to a better understanding of the risks and benefits of different therapeutic approaches applied to this group of patients.

In 2007, we published a European guideline for the management of bleeding following major trauma that included recommendations for specific interventions to identify and control bleeding sources using surgical, physiological and pharmacological strategies [13]. The guideline was developed by a multidisciplinary group of European experts, including designated representatives from relevant professional societies, to guide the clinician in the early phases of treatment. Here we present an updated version of the guideline that incorporates a renewed critical survey of the evidence published during the intervening three years and a consideration of changes in clinical practice that have taken place based on technologies that have become more widely available and pharmacological agents that have entered or left the market. Although the level of scientific evidence has improved in some areas, other areas remain devoid of high-level evidence, which may never exist for practical or ethical reasons. The formulation and grading of the recommendations presented here are therefore weighted to reflect both this reality and the current state-of-the-art.

## Materials and methods

These recommendations were formulated and graded according the Grading of Recommendations Assessment, Development and Evaluation (GRADE) hierarchy of evidence [14-16] summarised in Table 1. Comprehensive computer database literature searches were performed using the indexed online databases MEDLINE/PubMed and the Cochrane Library. Lists of cited literature within relevant articles were also screened. The primary intention of the review was to identify prospective randomised controlled trials (RCTs) and non-RCTs, existing systematic reviews and guidelines. In the absence of such evidence, case-control studies, observational studies and case reports were considered.

**Table 1:** Grading of recommendations from Guyatt and colleagues [14]

Grade of recommendation	Clarity of risk/benefit	Quality of supporting evidence	Implications
**1A**			
Strong recommendation, high-quality evidence	Benefits clearly outweigh risk and burdens, or vice versa	RCTs without important limitations or overwhelming evidence from observational studies	Strong recommendation, can apply to most patients in most circumstances without reservation
**1B**			
Strong recommendation, moderate-quality evidence	Benefits clearly outweigh risk and burdens, or vice versa	RCTs with important limitations (inconsistent results, methodological flaws, indirect or imprecise) or exceptionally strong evidence from observational studies	Strong recommendation, can apply to most patients in most circumstances without reservation
**1C**			
Strong recommendation, low-quality or very low-quality evidence	Benefits clearly outweigh risk and burdens, or vice versa	Observational studies or case series	Strong recommendation but may change when higher quality evidence becomes available
**2A**			
Weak recommendation, high-quality evidence	Benefits closely balanced with risks and burden	RCTs without important limitations or overwhelming evidence from observational studies	Weak recommendation, best action may differ depending on circumstances or patient or societal values
**2B**			
Weak recommendation, moderate-quality evidence	Benefits closely balanced with risks and burden	RCTs with important limitations (inconsistent results, methodological flaws, indirect or imprecise) or exceptionally strong evidence from observational studies	Weak recommendation, best action may differ depending on circumstances or patient or societal values
**2C**			
Weak recommendation, Low-quality or very low-quality evidence	Uncertainty in the estimates of benefits, risks, and burden; benefits, risk and burden may be closely balanced	Observational studies or case series	Very weak recommendation; other alternatives may be equally reasonable

Reprinted with permission from the American College of Chest Physicians.

RCTs, randomised controlled trials;

Boolean operators and Medical Subject Heading (MeSH) thesaurus keywords were applied as a standardised use of language to unify differences in terminology into single concepts. Appropriate MeSH headings and subheadings for each question were selected and modified based on search results. The scientific questions posed that led to each recommendation and the MeSH headings applied to each search are listed in Additional file 1. Searches were limited to English language abstracts and human studies, and gender and age were not limited. The time period was limited to the past three years for questions addressed in the 2007 version of the guideline, but no time-period limits were imposed on new searches. Original publications were evaluated for abstracts that were deemed relevant. Original publications were graded according to the levels of evidence developed by the Oxford Centre for Evidence-Based Medicine (Oxford, Oxfordshire, UK) [17].

The selection of the scientific enquiries to be addressed in the guideline, screening and grading of the literature to be included and formulation of specific recommendations were performed by members of the Task Force for Advanced Bleeding Care in Trauma, a multidisciplinary, pan-European group of experts with specialties in surgery, anaesthesia, emergency medicine, intensive care medicine and haematology. The core group was formed in 2004 to produce educational material on the care of the bleeding trauma patient [18], on which an update (in 2006) and subsequent review article were based [19]. The task force consisted of the core group, additional experts in haematology and guideline development, and representatives of relevant European professional societies, including the European Society of Anaesthesiology, the European Society of Intensive Care Medicine, the European Shock Society, the European Society of Trauma and Emergency Surgery and the European Society for Emergency Medicine. The European Hematology Association declined the invitation to designate a representative to join the task force.

As part of the guideline development process that led to the 2007 guideline, task force members participated in a workshop on the critical appraisal of medical literature. The nominal group process for the updated guideline included several remote (telephone and web-based) meetings and one face-to-face meeting supplemented by several Delphi rounds [20]. The guideline development group participated in a web conference in March 2009 to define the scientific questions to be addressed in the guideline. Selection, screening and grading of the literature and formulation of recommendations were accomplished in subcommittee groups consisting of at least three members via electronic or telephone communication. After distribution of the recommendations to the entire group, a face-to-face meeting of the task force was held in June 2009 with the aim of reaching a consensus on the draft recommendations from each subcommittee. After final refinement of the rationale for each recommendation and the complete manuscript, the updated document was approved by the endorsing organisations between October 2009 and January 2010. An updated version of the guideline is anticipated in due time.

In the GRADE system for assessing each recommendation, the letter attached to the grade of recommendation reflects the degree of literature support for the recommendation, whereas the number indicates the level of support for the recommendation assigned by the committee of experts. Recommendations are grouped by category and somewhat chronologically in the treatment decision-making process, but not by priority or hierarchy.

## Results

### I. Initial resuscitation and prevention of further bleeding

#### Minimal elapsed time

##### Recommendation 1

We recommend that the time elapsed between injury and operation be minimised for patients in need of urgent surgical bleeding control (Grade 1A).

##### Rationale

Trauma patients in need of emergency surgery for ongoing haemorrhage have increased survival if the elapsed time between the traumatic injury and admission to the operating theatre is minimised. More than 50% of all trauma patients with a fatal outcome die within 24 hours of injury [2]. Despite a lack of evidence from prospective RCTs, well-designed retrospective studies provide evidence for early surgical intervention in patients with traumatic haemorrhagic shock [21-23].

In addition, studies that analyse trauma systems indirectly emphasise the importance of minimising the time between admission and surgical bleeding control in patients with traumatic haemorrhagic shock [24,25]. At present, the evidence base for the impact of the implementation of the Advanced Trauma Life Support (ATLS) protocol on patient outcome is very poor, because the available literature focuses primarily on the effectiveness of ATLS as an educational tool [26]. Future studies are needed to define the impact of the ATLS program within trauma systems at the hospital and health system level in terms of controlled before-and-after implementation designed to assess post-injury mortality as the primary outcome parameter.

#### Tourniquet use

##### Recommendation 2

We recommend adjunct tourniquet use to stop life-threatening bleeding from open extremity injuries in the pre-surgical setting (Grade 1C).

##### Rationale

Much discussion has been generated recently regarding the use of tourniquets for acute external haemorrhage control. Pressure bandages rather than tourniquets should be applied in the case of minor bleeding from open wounds in extremity injuries. When uncontrolled arterial bleeding occurs from mangled extremity injuries, including penetrating or blast injuries or traumatic amputations, a tourniquet represents a simple and efficient method to acutely control haemorrhage [27-31]. Several publications from military settings report the effectiveness of tourniquets in this specific setting [27-30]. A study of volunteers showed that any tourniquet device presently on the market works efficiently [31]. The study also showed that 'pressure point control' was ineffective because collateral circulation was observed within seconds. Tourniquet-induced pain was not an important consideration.

Tourniquets should be left in place until surgical control of bleeding is achieved [28,30]; however, this time-span should be kept as short as possible. Improper or prolonged placement of a tourniquet can lead to complications such as nerve paralysis and limb ischaemia [32]. Some publications suggest a maximum time of application of two hours [32]. Reports from military settings report cases in which tourniquets have remained in place for up to six hours with survival of the extremity [28].

### II. Diagnosis and monitoring of bleeding

#### Initial assessment

##### Recommendation 3

We recommend that the physician clinically assess the extent of traumatic haemorrhage using a combination of mechanism of injury, patient physiology, anatomical injury pattern and the patient's response to initial resuscitation (Grade 1C).

##### Rationale

The mechanism of injury represents an important screening tool to identify patients at risk for significant traumatic haemorrhage. For example, the American College of Surgeons defined a threshold of 6 m (20 ft) as a 'critical falling height' associated with major injuries [33]. Further critical mechanisms include blunt versus penetrating trauma, high-energy deceleration impact, low-velocity versus high-velocity gunshot injuries, etc. The mechanism of injury in conjunction with injury severity, as defined by trauma scoring systems, and the patient's physiological presentation and response to resuscitation should further guide the decision to initiate early surgical bleeding control as outlined in the ATLS protocol [34-37]. Table 2 summarises estimated blood loss based on intitial presentation. Table 3 characterises the three types of response to initial fluid resuscitation, whereby the transient responders and the non-responders are candidates for immediate surgical bleeding control.

**Table 2:** American College of Surgeons Advanced Trauma Life Support (ATLS) classification of blood loss based on initial patient presentation

	Class I	Class II	Class III	Class IV
Blood loss* (ml)	Up to750	750-1500	1500-2000	>2000
Blood loss (% blood volume)	Up to 15%	15%-30%	30%-40%	>40%
Pulse rate	<100	100-120	120-140	>140
Blood pressure	Normal	Normal	Decreased	Decreased
Pulse pressure (mmHg)	Normal or increased	Decreased	Decreased	Decreased
Respiratory rate	14-20	20-30	30-40	>35
Urine output (ml/h)	>30	20-30	5-15	Negligible
Central nervous system/mental status	Slightly anxious	Mildly anxious	Anxious, confused	Confused, lethargic
Fluid replacement	Crystalloid	Crystalloid	Crystalloid and blood	Crystalloid and blood

Table reprinted with permission from the American College of Surgeons [37].

*for a 70 kg male

**Table 3:** American College of Surgeons Advanced Trauma Life Support (ATLS) responses to initial fluid resuscitation*

	Rapid response	Transient response	Minimal or no response
Vital signs	Return to normal	Transient improvement, recurrence of decreased blood pressure and increased heart rate	Remain abnormal
Estimated blood loss	Minimal (10%-20%)	Moderate and ongoing (20%-40%)	Severe (>40%)
Need for more crystalloid	Low	High	High
Need for blood	Low	Moderate to high	Immediate
Blood preparation	Type and crossmatch	Type-specific	Emergency blood release
Need for operative intervention	Possibly	Likely	Highly likely
Early presence of surgeon	Yes	Yes	Yes

* 2000 ml of isotonic solution in adults; 20 ml/kg bolus of Ringer's lactate in children.

Table reprinted with permission from the American College of Surgeons [37].

#### Ventilation

##### Recommendation 4

We recommend initial normoventilation of trauma patients if there are no signs of imminent cerebral herniation (Grade 1C).

##### Rationale

Ventilation can affect the outcome of severe trauma patients. There is a tendency for rescue personnel to hyperventilate patients during resuscitation [38,39], and hyperventilated trauma patients appear to have increased mortality when compared with non-hyperventilated patients [39].

A high percentage of severely injured patients with ongoing bleeding have traumatic brain injury (TBI). Relevant experimental and clinical data have shown that routine hyperventilation is an important contributor to adverse outcomes in patients with head injuries; however, the effect of hyperventilation on outcome in patients with severe trauma but no TBI is still a matter of debate. A low partial pressure of arterial carbon dioxide on admission to the emergency room is associated with a worse outcome in trauma patients with TBI [40-43].

There are several potential mechanisms for the adverse effects of hyperventilation and hypocapnia, including increased vasoconstriction with decreased cerebral blood flow and impaired tissue perfusion. In the setting of absolute or relative hypovolaemia, an excessive ventilation rate of positive-pressure ventilation may further compromise venous return and produce hypotension and even cardiovascular collapse [41,42]. It has also been shown that cerebral tissue lactic acidosis occurs almost immediately after induction of hypocapnia in children and adults with TBI and haemorrhagic shock [44]. In addition, even a modest level of hypocapnia (<27 mmHg) may result in neuronal depolarisation with glutamate release and extension of the primary injury via apoptosis [45].

Ventilation with low tidal volume is recommended in patients with acute lung injury. In patients with normal lung function, the evidence is scarce, but some observational studies show that the use of a high tidal volume is an important risk factor for the development of lung injury [46,47]. The injurious effect of high tidal volume may be initiated very early. Randomised studies demonstrate that short-time ventilation (<five hours) with high tidal volume (12 ml/kg) without positive end-expiratory pressure (PEEP) may promote pulmonary inflammation and alveolar coagulation in patients with normal lung function [48]. Although more studies are needed, the early use of protective ventilation with low tidal volume and moderate PEEP is recommended, particularly in bleeding trauma patients at risk of acute lung injury.

#### Immediate intervention

##### Recommendation 5

We recommend that patients presenting with haemorrhagic shock and an identified source of bleeding undergo an immediate bleeding control procedure unless initial resuscitation measures are successful (Grade 1B).

##### Rationale

The source of bleeding may be immediately obvious, and penetrating injuries are more likely to require surgical bleeding control. In a retrospective study of 106 abdominal vascular injuries, all 41 patients arriving in shock following gunshot wounds were candidates for rapid transfer to the operating theatre for surgical bleeding control [49]. A similar observation in a study of 271 patients undergoing immediate laparotomy for gunshot wounds indicates that these wounds combined with signs of severe hypovolaemic shock specifically require early surgical bleeding control. This observation is also true but to a lesser extent for abdominal stab wounds [50]. Data on injuries caused by penetrating metal fragments from explosives or gunshot wounds in the Vietnam War confirm the need for early surgical control when patients present in shock [51]. In blunt trauma, the mechanism of injury can determine to a certain extent whether the patient in haemorrhagic shock will be a candidate for surgical bleeding control. Only a few studies address the relation between the mechanism of injury and the risk of bleeding, and none of these publications is a randomised prospective trial of high evidence [52]. We have found no objective data describing the relation between the risk of bleeding and the mechanism of injury of skeletal fractures in general or of long-bone fractures in particular.

Traffic accidents are the leading cause of pelvic injury. Motor vehicle crashes cause approximately 60% of pelvic fractures followed by falls from great heights (23%). Most of the remainder result from motorbike collisions and vehicle-pedestrian accidents [53,54]. There is a correlation between 'unstable' pelvic fractures and intra-abdominal injuries [53,55]. An association between major pelvic fractures and severe head injuries, concomitant thoracic, abdominal, urological and skeletal injuries is also well described [53]. High-energy injuries produce greater damage to both the pelvis and organs. Patients with high-energy injuries require more transfusion units, and more than 75% have associated head, thorax, abdominal or genitourinary injuries [56]. It is well documented that 'unstable' pelvic fractures are associated with massive haemorrhage [55,57], and haemorrhage is the leading cause of death in patients with major pelvic fractures.

#### Further investigation

##### Recommendation 6

We recommend that patients presenting with haemorrhagic shock and an unidentified source of bleeding undergo immediate further investigation (Grade 1B).

##### Rationale

A patient in haemorrhagic shock with an unidentified source of bleeding should undergo immediate further assessment of the chest, abdominal cavity and pelvic ring, which represent the major sources of acute blood loss in trauma. Aside from a clinical examination, X-rays of chest and pelvis in conjunction with focused abdominal sonography for trauma (FAST) [58] or diagnostic peritoneal lavage (DPL) [59] are recommended diagnostic modalities during the primary survey [37,60,61]. In selected centres, readily available computed tomography (CT) scanners [62] may replace conventional radiographic imaging techniques during the primary survey.

#### Imaging

##### Recommendation 7

We recommend early imaging (FAST or CT) for the detection of free fluid in patients with suspected torso trauma (Grade 1B).

##### Recommendation 8

We recommend that patients with significant free intra-abdominal fluid and haemodynamic instability undergo urgent intervention (Grade 1A).

##### Recommendation 9

We recommend further assessment using CT for haemodynamically stable patients who are either suspected of having torso bleeding or have a high-risk mechanism of injury (Grade 1B).

##### Rationale

Blunt abdominal trauma represents a major diagnostic challenge and an important source of internal bleeding. FAST has been established as a rapid and non-invasive diagnostic approach for the detection of intra-abdominal free fluid in the emergency room [63-65]. Large prospective observational studies determined a high specificity and accuracy but low sensitivity of initial FAST examination for detecting intra-abdominal injuries in adults and children [66-72]. Liu and colleagues [73] found a high sensitivity, specificity and accuracy of initial FAST examination for the detection of haemoperitoneum. Although CT scans and DPL were shown to be more sensitive than sonography for the detection of haemoperitoneum, these diagnostic modalities are more time-consuming (CT and DPL) and invasive (DPL) [73].

The role of CT scanning of acute trauma patients is well documented [74-81], and in recent years imaging for trauma patients has migrated towards multi-slice CT (MSCT). The integration of modern MSCT scanners in the emergency room area allows the immediate assessment of trauma victims following admission [76,77]. Using modern MSCT scanners, total whole-body scanning time may be reduced to less than 30 seconds. In a retrospective study comparing 370 patients in two groups, Weninger and colleagues [77] showed that faster diagnosis using MSCT led to shorter emergency room and operating room time and shorter ICU stays [77]. Huber-Wagner and colleagues [62] also showed the benefit of integration of the whole-body CT into early trauma care. CT diagnosis significantly increases the probability of survival in patients with polytrauma. Whole-body CT as a standard diagnostic tool during the earliest resuscitation phase for polytraumatised patients provides the added benefit of identifying head and chest injuries and other bleeding sources in patients with multiple injuries.

Some authors have shown the benefit of contrast medium enhanced CT scanning. Anderson and colleagues [82,83] found high accuracy in the evaluation of splenic injuries resulting from trauma after administration of intravenous contrast material. Delayed phase CT may be used to detect active bleeding in solid organs. Fang and colleagues [84] demonstrated that the pooling of contrast material within the peritoneal cavity in blunt liver injuries indicates active and massive bleeding. Patients with this finding showed rapid deterioration of haemodynamic status and most of them required emergent surgery. Intraparenchymal pooling of contrast material with an unruptured liver capsule often indicates a self-limited haemorrhage, and these patients respond well to non-operative treatment.

Compared with MSCT, all traditional techniques of diagnostic and imaging evaluation are associated with some limitations. The diagnostic accuracy, safety and effectiveness of immediate MSCT are dependent on sophisticated pre-hospital treatment by trained and experienced emergency personnel and short transportation times [85,86]. If an MSCT is not available in the emergency room, the realisation of CT scanning implies transportation of the patient to the CT room, and therefore the clinician must evaluate the implications and potential risks and benefits of the procedure. During transport, all vital signs should be closely monitored and resuscitation measures continued. For those patients in whom haemodynamic stability is questionable, imaging techniques such as ultrasound and chest and pelvic radiography may be useful. Peritoneal lavage is rarely indicated if ultrasound or CT is available [87]. Transfer times to and from all forms of diagnostic imaging need to be considered carefully in any patient who is haemodynamically unstable. In addition to the initial clinical assessment, near patient testing results, including full blood count, haematocrit (Hct), blood gases and lactate, should be readily available under ideal circumstances.

Hypotensive patients (systolic blood pressure below 90 mmHg) presenting with free intra-abdominal fluid according to FAST or CT are potential candidates for early surgery if they cannot be stabilised by initiated fluid resuscitation [88-90]. A retrospective study by Rozycki and colleagues [91] of 1540 patients (1227 blunt, 313 penetrating trauma) assessed with FAST as an early diagnostic tool showed that the ultrasound examination had a sensitivity and specificity close to 100% when the patients were hypotensive.

A number of patients who present with free intra-abdominal fluid according to FAST can safely undergo further investigation with MSCT. Under normal circumstances, adult patients need to be haemodynamically stable when MSCT is performed outside of the emergency room [91]. Haemodynamically stable patients with a high risk mechanism of injury, such as high-energy trauma or even low-energy injuries in the elderly population, should be scanned after FAST for additional injuries using MSCT. As CT scanners are integrated in resuscitation units, whole-body CT diagnosis may replace FAST as a diagnostic method.

#### Haematocrit

##### Recommendation 10

We do not recommend the use of single Hct measurements as an isolated laboratory marker for bleeding (Grade 1B).

##### Rationale

Hct assays are part of the basic diagnostic work up for trauma patients. The diagnostic value of the Hct for detecting trauma patients with severe injury and occult bleeding sources has been a topic of debate in the past decade [92-94]. A major limit of the diagnostic value of Hct is the confounding influence of resuscitative measures on the Hct due to administration of intravenous fluids and red cell concentrates [94-97]. A retrospective study of 524 trauma patients determined a low sensitivity (0.5) of the initial Hct on admission for detecting those patients with traumatic haemorrhage requiring surgical intervention [94]. Two prospective observational diagnostic studies determined the sensitivity of serial Hct measurements for detecting patients with severe injury [92,93]. Decreasing serial Hct measurements may reflect continued bleeding, but the patient with significant bleeding may maintain his or her serial Hct.

#### Serum lactate and base deficit

##### Recommendation 11

We recommend both serum lactate and base deficit measurements as sensitive tests to estimate and monitor the extent of bleeding and shock (Grade 1B).

##### Rationale

Serum lactate has been used as a diagnostic parameter and prognostic marker of haemorrhagic shock since the 1960s [98]. The amount of lactate produced by anaerobic glycolysis is an indirect marker of oxygen debt, tissue hypoperfusion and the severity of haemorrhagic shock [99-102]. Similarly, base deficit values derived from arterial blood gas analysis provide an indirect estimation of global tissue acidosis due to impaired perfusion [99,101].

Vincent and colleagues [103] showed the value of serial lactate measurements for predicting survival in a prospective study in patients with circulatory shock. This study showed that changes in lactate concentrations provide an early and objective evaluation of a patient's response to therapy and suggested that repeated lactate determinations represent a reliable prognostic index for patients with circulatory shock [103]. Abramson and colleagues [104] performed a prospective observational study in patients with multiple trauma to evaluate the correlation between lactate clearance and survival. All patients in whom lactate levels returned to the normal range (≤2 mmol/l) within 24 hours survived. Survival decreased to 77.8% if normalisation occurred within 48 hours and to 13.6% in those patients in whom lactate levels were elevated above 2 mmol/l for more than 48 hours [104]. These findings were confirmed in a study by Manikis and colleagues [105] who showed that the initial lactate levels were higher in non-survivors after major trauma, and that the prolonged time for normalisation of lactate levels of more than 24 hours was associated with the development of post-traumatic organ failure [105].

Similar to the predictive value of lactate levels, the initial base deficit has been established as a potent independent predictor of mortality in patients with traumatic hemorrhagic shock [106]. Davis and colleagues [107] stratified the extent of base deficit into three categories, mild (-3 to -5 mEq/l), moderate (-6 to -9 mEq/l) and severe (<-10 mEq/l), and established a significant correlation between the admission base deficit and transfusion requirements within the first 24 hours and the risk of post-traumatic organ failure or death [107]. The same group of authors showed that the base deficit is a better prognostic marker of death than the pH in arterial blood gas analyses [108]. Furthermore, the base deficit was shown to represent a highly sensitive marker for the extent of post-traumatic shock and mortality, both in adult and paediatric patients [109,110].

In contrast to the data on lactate levels in haemorrhagic shock, reliable large-scale prospective studies on the correlation between base deficit and outcome are still lacking. Although both the base deficit and serum lactate levels are well correlated with shock and resuscitation, these two parameters do not strictly correlate with each other in severely injured patients [111]. Therefore, the independent assessment of both parameters is recommended for the evaluation of shock in trauma patients [99,101,111,112]. Composite scores that assess the likelihood of massive transfusion and include base deficit and other clinical parameters have been developed but require further validation [112,113]. Callaway and colleagues [114] performed a seven-year retrospective analysis of a prospective trauma registry from a level I trauma centre to determine predictors of mortality in elderly patients 65 years or older who sustained blunt trauma and presented with a normal initial systolic blood pressure (≥90 mmHg). The odds ratio for death was increased more than four-fold in those patients who had either elevated serum lactate levels above 4 mmol/l or a base deficit below -6 mEq/l, compared with patients with normal lactate levels (<2.5 mmol/l) or a base excess (>0 mEq/l). Paladino and colleagues [115] assessed the prognostic value of a combination of abnormal vital signs (heart rate >100 beats/min or a systolic blood pressure <90 mmHg) in conjunction with serum lactate and base deficit for identifying trauma patients with major injuries, using cut-off values for lactate at more than 2.2 mmol/l and base deficit at less than -2.0 mEq/l, respectively. The authors found that the addition of the metabolic parameters to the vital signs increased the sensitivity for identifying major injury from 40.9% to 76.4%, implying that the addition of lactate and base deficit to triage vital signs increases the ability to distinguish major from minor injury.

#### Coagulation monitoring

##### Recommendation 12

We recommend that routine practice to detect post-traumatic coagulopathy include the measurement of international normalised ratio (INR), activated partial thromboplastin time (APTT), fibrinogen and platelets. INR and APTT alone should not be used to guide haemostatic therapy (Grade 1C). We suggest that thrombelastometry also be performed to assist in characterising the coagulopathy and in guiding haemostatic therapy (Grade 2C).

##### Rationale

Little evidence supports a recommendation for the best haemostatic monitoring tool(s). Standard monitoring comprises INR, APTT, platelets and fibrinogen, although there is little direct evidence for the efficacy of these measures. Increasing emphasis focuses on the importance of fibrinogen and platelet measurements.

It is often assumed that the conventional coagulation screens (INR and APTT) monitor coagulation; however, these tests monitor only the initiation phase of blood coagulation and represent only the first 4% of thrombin production [116]. It is therefore possible that the conventional coagulation screen appears normal, while the overall state of blood coagulation is abnormal. Therefore, a more complete monitoring of blood coagulation and fibrinolysis, such as thrombelastometry, may facilitate more accurate targeting of therapy. Case series using thrombelastometry to assess trauma patients have been published. One study applied thrombelastometry to 23 patients, but without a comparative standard [117]. Another study found a poor correlation between thrombelastometry and conventional coagulation parameters [10]. Johansson [118] implemented a haemostatic resuscitation regime (early platelets and fresh frozen plasma (FFP)) guided using thrombelastometry in a before-and-after study which showed improved outcomes. There is insufficient evidence at present to support the utility of thrombelastometry in the detection of post-traumatic coagulopathy. More research is required in this area, and in the meantime physicians should make their own judgement when developing local policies.

It is theoretically possible that the pattern of change in measures of coagulation such as D-dimers may help to identify patients with ongoing bleeding. However, there are no publications relevant to this question, so traditional methods of detection for ongoing bleeding, such as serial clinical evaluation of radiology (ultrasound, CT or angiography) should be used.

### III. Rapid control of bleeding

#### Pelvic ring closure and stabilisation

##### Recommendation 13

We recommend that patients with pelvic ring disruption in haemorrhagic shock undergo immediate pelvic ring closure and stabilisation (Grade 1B).

#### Packing, embolisation and surgery

##### Recommendation 14

We recommend that patients with ongoing haemodynamic instability despite adequate pelvic ring stabilisation receive early preperitoneal packing, angiographic embolisation and/or surgical bleeding control (Grade 1B).

##### Rationale

The mortality rate of patients with severe pelvic ring disruptions and haemodynamic instability remains unacceptably high [119-122]. The early detection of these injuries and initial efforts to reduce disruption and stabilise the pelvis as well as containing bleeding is therefore crucial. Markers of pelvic haemorrhage include anterior-posterior and vertical shear deformations, CT 'blush' (active arterial extravasation), bladder compression pressure, pelvic haematoma volumes of more than 500 ml evident by CT and ongoing haemodynamic instability despite adequate fracture stabilisation [123-125].

The initial therapy of pelvic fractures includes control of venous and/or cancellous bone bleeding by pelvic closure. Some institutions use primarily external fixators to control haemorrhage from pelvic fractures [124,125] but pelvic closure may also be achieved using a bed sheet, pelvic binder or a pelvic C-clamp [126-128]. In addition to the pelvic closure, fracture stabilisation and the tamponade effect of the haematoma, pre, extra or retroperitoneal packing will reduce or stop the venous bleeding [122,129-131]. Preperitoneal packing decreases the need for pelvic embolisation and may be performed simultaneously or soon after initial pelvic stabilisation [122,129,131]. The technique can be combined with a consecutive laparotomy if deemed necessary [122,129]. This may decrease the high mortality rate observed in patients with major pelvic injuries who underwent laparotomy as the primary intervention. As a consequence, it was recommended that non-therapeutic laparotomy should be avoided [132].

Angiography and embolisation is currently accepted as a highly effective means with which to control arterial bleeding that cannot be controlled by fracture stabilisation [122-126,131-140]. The presence of sacroiliac joint disruption, female gender and duration of hypotension can reliably predict patients who would benefit from the procedure [138]. Controversy exists about the indications and optimal timing of angiography in haemodynamically unstable patients [131]. Institutional differences in the capacity to perform timely angiography and embolisation may explain the different treatment algorithms suggested by many authors [119-122,125,129,131,132,140]. Nevertheless, the general consensus is that a multidisciplinary approach to these severe injuries is required.

#### Early bleeding control

##### Recommendation 15

We recommend that early bleeding control of the abdomen be achieved using packing, direct surgical bleeding control and the use of local haemostatic procedures. In the exsanguinating patient, aortic cross-clamping may be employed as an adjunct (Grade 1C).

##### Rationale

Abdominal resuscitative packing is an early part of the post-traumatic laparotomy to identify major injuries and sources of haemorrhage [141,142]. If bleeding cannot be controlled using packing and conventional surgical techniques when the patient is in extremis or when proximal vascular control is deemed necessary before opening the abdomen, aortic cross clamping may be employed as an adjunct to reduce bleeding and redistribute blood flow to the heart and brain [143-145]. When blood losses are important, when surgical measures are unsuccessful and/or when the patient is cold, acidotic and coagulopathic, definitive packing may also be the first surgical step within the concept of damage control [146-155]. Packing aims to compress liver ruptures or exert direct pressure on the sources of bleeding [141,142,146-150,152-154]. The definitive packing of the abdomen may allow further attempts to achieve total haemostasis through angiography and/or correction of coagulopathy [155]. The removal of packs should preferably be performed only after 48 hours to lower the risk of rebleeding [152,153].

#### Damage control surgery

##### Recommendation 16

We recommend that damage control surgery be employed in the severely injured patient presenting with deep haemorrhagic shock, signs of ongoing bleeding and coagulopathy. Additional factors that should trigger a damage control approach are hypothermia, acidosis, inaccessible major anatomical injury, a need for time-consuming procedures or concomitant major injury outside the abdomen (Grade 1C).

##### Rationale

The severely injured patient arriving to the hospital with continuous bleeding or deep haemorrhagic shock generally has a poor chance of survival unless early control of bleeding, proper resuscitation and blood transfusion are achieved. This is particularly true for patients who present with uncontrolled bleeding due to multiple penetrating injuries or patients with multiple injuries and unstable pelvic fractures with ongoing bleeding from fracture sites and retroperitoneal vessels. The common denominator in these patients is the exhaustion of physiological reserves with resulting profound acidosis, hypothermia and coagulopathy, also known as the 'bloody vicious cycle'. In 1983, Stone and colleagues described the techniques of abbreviated laparotomy, packing to control haemorrhage and of deferred definitive surgical repair until coagulation has been established [156]. Since then, a number of authors have described the beneficial results of this concept, now called 'damage control' [50,54,121,134,151,156-158]. Damage control surgery of the abdomen consists of three components: the first component is an abbreviated resuscitative laparotomy for control of bleeding, the restitution of blood flow where necessary and the control of contamination. This should be achieved as rapidly as possible without spending unnecessary time on traditional organ repairs that can be deferred to a later phase. The abdomen is packed and temporary abdominal closure is performed. The second component is intensive care treatment, focused on core re-warming, correction of the acid-base imbalance and coagulopathy as well as optimising the ventilation and the haemodynamic status. The third component is the definitive surgical repair that is performed only when target parameters have been achieved [159-162]. Although the concept of 'damage control' intuitively makes sense, no RCTs exist to support it. Retrospective studies support the concept showing reduced morbidity and mortality rates in selective populations [50,151,157,161].

The same 'damage control' principles have been applied to orthopaedic injuries in severely injured patients [134,163-166]. Scalea was the first to coin the term 'damage control orthopaedics' [166]. Relevant fractures are primarily stabilised with external fixators rather than primary definitive osteosynthesis [134,163]. The less traumatic and shorter duration of the surgical procedure aims to reduce the secondary trauma load. Definitive osteosynthesis surgery can be performed after 4 to 14 days when the patient has recovered sufficiently. Retrospective clinical studies and prospective cohort studies seem to support the concept of damage control [134,163-165]. The only available randomised study shows an advantage for this strategy in 'borderline' patients [164].

#### Local haemostatic measures

##### Recommendation 17

We recommend the use of topical haemostatic agents in combination with other surgical measures or with packing for venous or moderate arterial bleeding associated with parenchymal injuries (Grade 1B).

##### Rationale

A wide range of local haemostatic agents are currently available for use as adjuncts to traditional surgical techniques to obtain haemorrhage control. These topical agents can be particularly useful when access to the bleeding area is difficult. Local haemostatic agents include collagen, gelatin or cellulose-based products, fibrin and synthetic glues or adhesives that can be used for both external and internal bleeding while polysaccharide-based and inorganic haemostatics are still mainly used and approved for external bleeding. The use of topical haemostatic agents should consider several factors such as the type of surgical procedure, cost, severity of bleeding, coagulation status and each agent's specific characteristics. Some of these agents should be avoided when autotransfusion is used and several other contraindications need to be considered [167,168]. The capacity of each agent to control bleeding was initially studied in animals but increasing experience from humans is now available [167-180].

The different types of local haemostatics are briefly presented according to their basis and haemostatic capacity:

i) Collagen-based agents trigger platelet aggregation resulting in clot formation when in contact with a bleeding surface. They are often combined with a procoagulant substance such as thrombin to enhance the haemostatic effect. A positive haemostatic effect has been shown in several human studies [169-172].

ii) Gelatin-based products can be used alone or in combination with a procoagulant substance [167]. Swelling of the gelatin in contact with blood reduces the blood flow and, in combination with a thrombin-based component, enhances haemostasis. A similar or superior haemostatic effect has been observed compared with collagen-based agents [173-175].

iii) The effect of cellulose-based haemostatic agents on bleeding has been less well studied and only case reports that support their use are available.

iv) Fibrin and synthetic glues or adhesives have both haemostatic and sealant properties and their significant effect on haemostasis have been shown in several human RCTs involving vascular, bone, skin and visceral surgery [176-178].

v) Polysaccharide-based haemostatics can be divided into two broad categories [167]: N-acetyl-glucosamine-containing glycosaminoglycans purified from microalgae and diatoms and microporous polysaccharide haemospheres produced from potato starch. The mechanism of action is complex and depends on the purity or combination with other substances such as cellulose or fibrin. A number of different products are currently available and have been shown to be efficient for external use. An observational study showed that haemorrhage control was achieved using an N-acetylglucosamine-based bandage applied to 10 patients with severe hepatic and abdominal injuries, acidosis and clinical coagulopathy [180].

vi) The inorganic haemostatics based on minerals such as zeolite or smectite have been used and studied mainly on external bleeding [167,168].

### IV. Tissue oxygenation, fluid and hypothermia

#### Volume replacement

##### Recommendation 18

We recommend a target systolic blood pressure of 80 to 100 mmHg until major bleeding has been stopped in the initial phase following trauma without brain injury (Grade 1C).

##### Rationale

In order to maintain tissue oxygenation, traditional treatment of trauma patients uses early and aggressive fluid administration to restore blood volume. This approach may, however, increase the hydrostatic pressure on the wound, cause a dislodgement of blood clots, a dilution of coagulation factors and undesirable cooling of the patient. The concept of low-volume fluid resuscitation, so-called 'permissive hypotension', avoids the adverse effects of early aggressive resuscitation while maintaining a level of tissue perfusion that, although lower than normal, is adequate for short periods [130]. A controlled hypotensive fluid resuscitation should aim to achieve a mean arterial pressure of 65 mmHg or more [181]. Its general effectiveness remains to be confirmed in RCTs; however, studies have demonstrated increased survival when a low volume fluid resuscitation concept was used in penetrating trauma [182,183]. In contrast, no significant difference in survival was found in patients with blunt trauma [184]. One study concluded that mortality was higher after on-site resuscitation compared with in-hospital resuscitation [185]. It seems that greater increases in blood pressure are tolerated without exacerbating haemorrhage when they are achieved gradually and with a significant delay following the initial injury [186]. All the same, a recent Cochrane systematic review concluded that there is no evidence from RCTs for or against early or larger volume intravenous fluids to treat uncontrolled haemorrhage [187]. However, a recent retrospective analysis demonstrated that aggressive resuscitation techniques, often initiated in the prehospital setting, appear to increase the likelihood that patients with severe extremity injuries develop secondary abdominal compartment syndrome (ACS) [188]. In this study, early, large-volume crystalloid administration was the greatest predictor of secondary ACS. Moreover, a retrospective analysis of the German Trauma Registry database including 17,200 multiply injured patients showed that the incidence of coagulopathy increased with increasing volume of intravenous fluids administered pre-clinically. Coagulopathy was observed in more than 40% of patients with more than 2000 ml, in more than 50% with more than 3000 ml, and in more than 70% with more than 4000 ml administered [3].

The low-volume approach is contraindicated in TBI and spinal injuries, because an adequate perfusion pressure is crucial to ensure tissue oxygenation of the injured central nervous system. In addition, the concept of permissive hypotension should be carefully considered in the elderly patient and may be contraindicated if the patient suffers from chronic arterial hypertension.

A recent analysis from an ongoing multi-centre prospective cohort study suggests that the early use of vasopressors for haemodynamic support after haemorrhagic shock in comparison to aggressive volume resuscitation may be deleterious and should be used cautiously [189]. However, this study has several limitations: the study is a secondary analysis of a prospective cohort study, and was not designed to answer the specific hypothesis tested. Thus, it is not possible to separate vasopressor from the early management of trauma patients. In addition, although the use of a vasopressor helps to rapidly restore arterial pressure, it should not be viewed as a substitute for fluid resuscitation and the target blood pressure must be respected.

#### Fluid therapy

##### Recommendation 19

We recommend that crystalloids be applied initially to treat the bleeding trauma patient (Grade 1B). We suggest that hypertonic solutions also be considered during initial treatment (Grade 2B). We suggest that the addition of colloids be considered within the prescribed limits for each solution in haemodynamically unstable patients (Grade 2C).

##### Rationale

It is still unclear what type of fluid should be employed in the initial treatment of the bleeding trauma patient. Although several meta-analyses have shown an increased risk of death in patients treated with colloids compared with patients treated with crystalloids [190-194] and three of these studies showed that the effect was particularly significant in a trauma subgroup [190,193,194], a more recent meta-analysis showed no difference in mortality between colloids and crystalloids [195]. If colloids are used, modern hydroxyethyl starch or gelatin solutions should be used because the risk:benefit ratio of dextran is disadvantageous. Problems in evaluating and comparing the use of different resuscitation fluids include the heterogeneity of populations and therapy strategies, limited quality of analysed studies, mortality not always being the primary outcome, and different, often short, observation periods. It is therefore difficult to reach a definitive conclusion as to the advantage of one type of resuscitation fluid over the other. The Saline versus Albumin Fluid Evaluation study compared 4% albumin with 0.9% sodium chloride in 6997 ICU patients and showed that albumin administration was not associated with worse outcomes; however, there was a trend towards higher mortality in the brain trauma subgroup that received albumin (*P *= 0.06) [196]. Promising results have been obtained with hypertonic solutions. Recently, a double-blind, RCT in 209 patients with blunt traumatic injuries analysed the effect of the treatment with 250 ml of 7.5% hypertonic saline and 6% dextran 70 compared with lactated Ringer solution on organ failure. The intent-to-treat analysis demonstrated no significant difference in organ failure and in acute respiratory disress syndrome (ARDS)-free survival. However, there was improved ARDS-free survival in the subset (19% of the population) requiring 10 U or more of packed red blood cells (RBCs) [197]. One study showed that the use of hypertonic saline was associated with lower intracranial pressure than with normal saline in brain-injured patients [198] and a meta-analysis comparing hypertonic saline dextran with normal saline for resuscitation in hypotension from penetrating torso injuries showed improved survival in the hypertonic saline dextran group when surgery was required [199]. A clinical trial with brain injury patients found that hypertonic saline reduced intracranial pressure more effectively than dextran solution with 20% mannitol when compared in equimolar dosing [200]. However, Cooper and colleagues found almost no difference in neurological function six months after TBI in patients who had received pre-hospital hypertonic saline resuscitation compared with conventional fluid [201]. In conclusion, the evidence suggests that hypertonic saline solutions are safe, and will improve haemodynamics during hypovolaemic resuscitation. The evidence for increased survival with use of hypertonic saline solutions is inconclusive. It is possible that certain subgroups might benefit from hypertonic saline solutions, but further research is required [202].

#### Normothermia

##### Recommendation 20

We recommend early application of measures to reduce heat loss and warm the hypothermic patient in order to achieve and maintain normothermia (Grade 1C).

##### Rationale

Hypothermia, defined as a core body temperature below 35°C, is associated with acidosis, hypotension and coagulopathy in severely injured patients. In a retrospective study with 122 patients, hypothermia was an ominous clinical sign, accompanied by high mortality and blood loss [203]. The profound clinical effects of hypothermia ultimately lead to higher morbidity and mortality, and hypothermic patients require more blood products [204].

Hypothermia is associated with an increased risk of severe bleeding, and hypothermia in trauma patients represents an independent risk factor for bleeding and death [205]. The effects of hypothermia include altered platelet function, impaired coagulation factor function (a 1°C drop in temperature is associated with a 10% drop in function), enzyme inhibition and fibrinolysis [206,207]. Body temperatures below 34°C compromise blood coagulation, but this has only been observed when coagulation tests (prothrombin time (PT) and APTT) are carried out at the low temperatures seen in patients with hypothermia, and not when assessed at 37°C as is routine practice for such tests. Steps to prevent hypothermia and the risk of hypothermia-induced coagulopathy include removing wet clothing, covering the patient to avoid additional heat loss, increasing the ambient temperature, forced air warming, warm fluid therapy and, in extreme cases, extracorporeal re-warming devices [208,209].

Animal and human studies of controlled hypothermia in haemorrhage have shown some positive results compared with normothermia [210,211]. Contradictory results have been observed in meta-analyses that examine mortality and neurological outcomes associated with mild hypothermia in patients with TBI, possibly due to the different exclusion and inclusion criteria for the studies used for the analysis [212-214]. The speed of induction and duration of hypothermia, which may be very important factors that influence the benefit associated with this treatment. It has been shown that five days of long-term cooling is more efficacious than two days of short-term cooling when mild hypothermia is used to control refractory intracranial hypertension in adults with severe TBI [215]. Obviously, the time span of hypothermia is crucial, because a recent prospective RCT in 225 children with severe TBI showed that hypothermic therapy initiated within 8 hours after injury and continued for 24 hours did not improve the neurological outcome and may increase mortality [216]. Furthermore, the mode of inducing cerebral hypothermia induction may influence its effectiveness. In a RCT comparing non-invasive selective brain cooling (33 to 35°C) in 66 patients with severe TBI and mild systemic hypothermia (rectal temperature 33 to 35°C) and a control group not exposed to hypothermia, natural rewarming began after three days. Mean intracranial pressure 24, 48 or 72 hours after injury was significantly lower in the selective brain cooling group than in the control group [217].

Prolonged hypothermia may be considered in patients with isolated head trauma after haemorrhage has been arrested. If mild hypothermia is applied in TBI, cooling should take place within the first three hours following injury, preferably using selective brain cooling by cooling the head and neck, be maintained for at least 48 hours [218], rewarming should last 24 hours and the cerebral perfusion pressure should be maintained above 50 mmHg (systolic blood pressure ≥70 mmHg). Patients most likely to benefit from hypothermia are those with a GCS at admission between 4 and 7 [219]. Possible side effects are hypotension, hypovolaemia, electrolyte disorders, insulin resistance and reduced insulin secretion and increased risk of infection [220]. Further studies are warranted to investigate the postulated benefit of hypothermia in TBI taking these important factors into account.

### V. Management of bleeding and coagulation

#### Erythrocytes

##### Recommendation 21

We recommend a target haemoglobin (Hb) of 7 to 9 g/dl (Grade 1C).

##### Rationale

Erythrocytes contribute to haemostasis by influencing the biochemical and functional responsiveness of activated platelets via the rheological effect on platelet margination and by supporting thrombin generation [221]; however, the optimal Hct or Hb concentration required to sustain haemostasis in massively bleeding patients is unclear. Further investigations into the role of the Hb concentration on haemostasis in massively transfused patients are therefore warranted.

The effects of the Hct on blood coagulation have not been fully elucidated [222]. An acute reduction of the Hct results in an increase in the bleeding time [223,224] with restoration upon re-transfusion [223]. This may relate to the presence of the enzyme elastase on the surface of RBC membranes, which may activate coagulation factor IX [225,226]. However, a moderate reduction of the Hct does not increase blood loss from a standard spleen injury [224], and an isolated *in vitro *reduction of the Hct did not compromise blood coagulation as assessed by thrombelastometry [227].

No prospective RCT has compared restrictive and liberal transfusion regimens in trauma, but 203 trauma patients from the Transfusion Requirements in Critical Care trial [228] were re-analysed [229]. A restrictive transfusion regimen (Hb transfusion trigger <7.0 g/dl) resulted in fewer transfusions as compared with the liberal transfusion regimen (Hb transfusion trigger <10 g/dl) and appeared to be safe. However, no statistically significant benefit in terms of multiple organ failure or post-traumatic infections was observed. It should be emphasised that this study was neither designed nor powered to answer these questions with precision. In addition, it cannot be ruled out that the number of RBC units transfused merely reflects the severity of injury. Nevertheless, RBC transfusions have been shown in multiple studies to be associated with increased mortality [230-234], lung injury [234-236], increased infection rates [237,238] and renal failure in trauma victims [233]. This ill effect may be particularly important with RBC transfusions stored for more than 14 days [233].

Despite the lack of high-level scientific evidence for a specific Hb transfusion trigger in patients with TBI, these patients are currently transfused in many centres to achieve an Hb of approximately 10 g/dl [239]. This might be justified by the recent finding that increasing the Hb from 8.7 to 10.2 g/dl improved local cerebral oxygenation in 75% of patients [158]. In another preliminary study in patients with TBI, one to two RBC transfusions at a Hb of approximately 9 g/dl transiently (three to six hours) increased cerebral oxygenation, again in approximately 75% of patients [240,241]. A storage time of more than 19 days precluded this effect [240]. In another recent study, cerebral tissue oxygenation, on average, did not increase due to an increase in Hb from 8.2 to 10.1 g/dl [242]. Nevertheless, the authors came to the conclusion based on multivariable statistical models that the changes in cerebral oxygenation correlated significantly with Hb concentration [242]. This conclusion, however, was questioned in the accompanying editorial [243].

In an initial outcome study the lowest Hct was correlated with adverse neurological outcome and RBC transfusions were also found to be an independent factor predicting adverse neurological outcome [244]. Interestingly, the number of days with a Hct below 30% was found to be correlated with an improved neurological outcome [244]. In a more recent outcome study in 1150 patients with TBI, RBC transfusions were found to be associated with a two-fold increased mortality and a three-fold increased complication rate [138]. Therefore, patients with severe TBI should not have an Hb transfusion threshold different than that of other critically ill patients.

#### Coagulation support

##### Recommendation 22

We recommend that monitoring and measures to support coagulation be initiated as early as possible (Grade 1C).

##### Rationale

Major trauma results not only in bleeding from anatomical sites but also frequently in coagulopathy, which is associated with a several-fold increase in mortality [3,5,8,9,245]. This early coagulopathy of trauma is mainly found in patients with hypoperfusion (base deficit >6 mE/l) [8,245] and is characterised by an up-regulation of endothelial thrombomodulin, which forms complexes with thrombin [246].

Early monitoring of coagulation is essential to detect trauma-induced coagulopathy and to define the main causes, including hyperfibrinolysis [10,117]. Early therapeutic intervention does improve coagulation tests [247] and persistent coagulopathy at ICU entry has been shown to be associated with a increased mortality [248]. Therefore, early aggressive treatment is likely to improve the outcome of severely injured patients [249]. However, there are also studies in which no survival benefit could be shown [247,250].

#### Calcium

##### Recommendation 23

We recommend that ionised calcium levels be monitored during massive transfusion (Grade 1C). We suggest that calcium chloride be administered during massive transfusion if ionised calcium levels are low or electrocardiographic changes suggest hypocalcaemia (Grade 2C).

##### Rationale

Calcium in the extracellular plasma exists either in a free ionised state (45%) or bound to proteins and other molecules in a biologically inactive state (55%). The normal concentration of the ionised form ranges from 1.1 to 1.3 mmol/l and is influenced by the pH. A 0.1 unit increase in pH decreases the ionised calcium concentration by approximately 0.05 mmol/l [181]. The availability of ionised calcium is essential for the timely formation and stabilisation of fibrin polymerisation sites, and a decrease in cytosolic calcium concentration precipitates a decrease in all platelet-related activities [181]. In addition, contractility of the heart and systemic vascular resistance are compromised at low ionised calcium levels. Combining beneficial cardiovascular and coagulation effects, the level for ionised calcium concentration should therefore be maintained above 0.9 mmol/l [181].

Early hypocalcaemia following traumatic injury shows a significant correlation with the amount of infused colloids, but not with crystalloids, and may be attributable to colloid-induced haemodilution [251]. Also, hypocalcaemia develops during massive transfusion as a result of the citrate employed as an anticoagulant in blood products. Citrate exerts its anticoagulant activity by binding ionised calcium, and hypocalcaemia is most common in association with FFP and platelet transfusion because these products contain high citrate concentrations. Citrate undergoes rapid hepatic metabolism, and hypocalcaemia is generally transient during standard transfusion procedures. Citrate metabolism may be dramatically impaired by hypoperfusion states, hypothermia and in patients with hepatic insufficiency [252].

#### Fresh frozen plasma

##### Recommendation 24

We recommend early treatment with thawed FFP in patients with massive bleeding (Grade 1B). The initial recommended dose is 10 to 15 ml/kg. Further doses will depend on coagulation monitoring and the amount of other blood products administered (Grade 1C).

##### Rationale

The clinical efficacy of FFP is largely unproven [253]. Nevertheless, most guidelines recommend the use of FFP either in massive bleeding or in significant bleeding complicated by coagulopathy (PT or APTT more than 1.5 times control) [7,254,255]. Patients treated with oral anticoagulants (vitamin K antagonists) present a particular challenge, and FFP is recommended [255] only when prothrombin complex concentrate (PCC) is not available [254]. The most frequently recommended dose is 10 to 15 ml/kg [254,255], and further doses may be required [256]. As with all products derived from human blood, the risks associated with FFP treatment include circulatory overload, ABO incompatibility, transmission of infectious diseases (including prion diseases), mild allergic reactions and transfusion-related acute lung injury [254,257,258]. FFP and platelet concentrates appear to be the most frequently implicated blood products in transfusion-related acute lung injury [257-260]. Although the formal link between the administration of FFP, control of bleeding and an eventual improvement in the outcome of bleeding patients is lacking, most experts would agree that FFP treatment is beneficial in patients with massive bleeding or significant bleeding complicated by coagulopathy.

There are very few well-designed studies that explore massive transfusion strategy. The need for massive transfusion is relatively rare, occurring in less than 2% of civilian trauma patients, but higher (7%) in the military setting. Massive transfusion management has been based on the concept that coagulopathy associated with severe trauma was primarily consumptive due to the dilution of blood clotting factors and the consumption of haemostasis factors at the site of injury.

FFP was recommended when PT or APTT was 1.5 times normal or after 10 RBC units had been transfused. Many massive transfusion protocols stipulated one unit of FFP for every four units of RBCs. In recent years, retrospective data from the US Army combat support hospitals have shown an association between survival and a higher ratio of transfused FFP and RBC units. These data show that casualties who received FFP and RBCs at a ratio of 1:4 or lower, had a three-fold higher mortality than those who received a massive transfusion with a 2:3 ratio. These data have induced many civilian trauma centres to modify their transfusion approach to incorporate the early use of thawed FFP in ratios approaching 1:1.

Ten relevant studies addressing FFP:RBC ratio have been identified, all of which were retrospective studies, although some are based on data collected prospectively for other reasons. None of the studies were clinical RCTs. The majority of the authors used massive transfusion (10 RBC units within 24 hours) as the entry criterion; however, to limit bias due to FFP unavailability, one study [261] excluded patients who died within the first 30 minutes. One of the studies [262] took into consideration only patients alive upon ICU admission, and another defined massive transfusion as 10 units or more prior to ICU admission. One report [247] defined massive transfusion as more than 10 units over 6 hours. Two of the studies are based on data collected in a combat setting, while the other eight were performed based on data collected at civilian trauma centres. The majority of the studies are single centre; one study is multi-centre [261] and one is a retrospective analysis of the German Trauma Registry [3].

Seven studies showed better outcomes using a high FFP:RBC ratio [3,261-266] and two did not [250,267]. One study may be classified as indeterminate because a high FFP:RBC ratio (average 1:2) was associated with a better survival than a low ratio (average 1:4), but the survival curve was U-shaped, with the lowest mortality at a 1:2 to 1:3 ratio [247]. The two combat studies showed better outcomes using a high ratio [265,266]. Early empirical infusion of FFP may increase the frequency of delayed traumatic intracerebral haematoma and the mortality in patients with severe head injury [268]. Most of the studies calculate FFP:RBC ratio at 24 hours after admission. When Snyder and colleagues [267] used the FFP:RBC ratio at 24 hours as a fixed value, patients who received a higher ratio had significantly better outcomes, but if the timing of component product transfusion was taken into account, the difference was no longer statistically significant.

These combat data are retrospective, refer to young, previously healthy male patients with penetrating injuries and may be confounded to some extent by treatment biases. Because FFP requires a significant amount of time before it is thawed and available for transfusion and many trauma deaths occur soon after hospital admission, patients who die early may receive RBC units but die before FFP therapy has begun. These cases may therefore be included in the low ratio group even if a 1:1 strategy was intended. One further ground for criticism of many of these studies is that the number of RBCs units transfused is an indicator of severity of injury that cannot be completely adjusted for by regression analysis. All of these limitations must be kept in mind when analysing the available recent literature and emphasises the need for prospective trials.

#### Platelets

##### Recommendation 25

We recommend that platelets be administered to maintain a platelet count above 50 × 10^9^/l (Grade 1C). We suggest maintenance of a platelet count above 100 × 10^9^/l in patients with multiple trauma who are severely bleeding or have TBI (Grade 2C). We suggest an initial dose of four to eight platelet concentrates or one aphaeresis pack (Grade 2C).

##### Rationale

In medical conditions leading to thrombocytopaenia, haemorrhage does not often occur until the platelet count falls below 50 × 10^9^/l, and platelet function decreases exponentially below this point [269-272]. There is no direct evidence to support a particular platelet transfusion threshold in the trauma patient. A consensus development conference sponsored by the National Institutes of Health (NIH; Bethesda, MD, USA) in 1986 determined that bleeding is unlikely to be caused by thrombocytopaenia at platelet counts of 50 × 10^9^/l or greater and agreed that platelet transfusion is appropriate to prevent or control bleeding associated with deficiencies in platelet number or function [273,274]. The NIH consensus did not consider trauma, but it seems reasonable to recommend that a platelet count of at least 50 × 10^9^/l be maintained following injury.

An argument can be made for maintaining a higher level of platelets, perhaps up to 100 × 10^9^/l, following injury. If a patient has increased fibrin degradation products due to disseminated intravascular coagulation and/or hyperfibrinolysis, this will interfere with platelet function and a higher threshold of 75 × 10^9^/l has been suggested by consensus groups [275,276]. Moreover, platelet-rich concentrate is an autologous concentration of platelets and growth factors (e.g. transforming growth factor-beta, vascular endothelial growth factor and platelet-derived growth factor), and due to the increased concentration and release of these factors, platelet-rich concentrates could potentially enhance bone and soft tissue healing [277]. Transfusion threshold levels of up to 100 × 10^9^/l have been suggested for treatment of severe brain injury and massive haemorrhage, but the evidence for the higher threshold is weak [275,276]. One group showed that trauma patients receiving platelets and RBCs at a ratio of 1:5 or greater had a lower 30-day mortality when compared with those with who received less than this ratio (38% vs. 61%, *P *= 0.001) [264]. Another study of massively transfused trauma patients has pointed to an early aggressive correction of coagulopathy with platelet transfusion as a possible contributing factor to good outcome [278]. In this retrospective cohort study, survivors received one platelet transfusion for every 7.7 units of blood transfused whereas nonsurvivors received only one platelet transfusion for every 11.9 units of blood transfused (*P *= 0.03).

When platelet transfusion was introduced in the 1950s, no clinical trials were employed to assess the utility of platelet therapy compared with placebo, and such trials today might be considered unethical. The appropriate dose of platelets is therefore uncertain. Platelet concentrate produced from a unit of whole blood contains 7.5 × 10^10 ^platelets on average and should increase the platelet count by 5 to 10 × 10^9^/l in a 70 kg recipient. Aphaeresis platelet concentrates generally contain approximately 3 to 6 × 10^11 ^platelets, depending on local collection practice, and physicians should be cognisant of the doses provided locally. A pool of four to eight platelet concentrates or a single-donor aphaeresis unit is usually sufficient to provide haemostasis in a thrombocytopaenic, bleeding patient. If required, the dose of platelets (× 10^9^) can be calculated in more detail from the desired platelet increment, the patient's blood volume in litres (estimated by multiplying the patient's body surface area by 2.5, or 70 ml/kg in an adult), and a correction factor of 0.67 to allow for pooling of approximately 33% of transfused platelets in the spleen.

#### Fibrinogen and cryoprecipitate

##### Recommendation 26

We recommend treatment with fibrinogen concentrate or cryoprecipitate if significant bleeding is accompanied by thrombelastometric signs of a functional fibrinogen deficit or a plasma fibrinogen level of less than 1.5 to 2.0 g/l (Grade 1C). We suggest an initial fibrinogen concentrate dose of 3 to 4 g or 50 mg/kg of cryoprecipitate, which is approximately equivalent to 15 to 20 units in a 70 kg adult. Repeat doses may be guided by thrombelastometric monitoring and laboratory assessment of fibrinogen levels (Grade 2C).

##### Rationale

The formation of fibrin is a key step in blood coagulation [222,279], and hypofibrinogenemia is a usual component of complex coagulopathies associated with massive bleeding. Coagulopathic civilian trauma patients had a fibrinogen concentration of 0.9 g/l (interquartile ratio (IQR) 0.5 to 1.5 g/l) in conjunction with a maximum clot firmness of 6 mm (IQR 0 to 9 mm) using thrombelastometry, whereas only 2.5% of healthy volunteers had a maximum clot firmness of 7 mm or less [10]. In trauma patients, a maximum clot firmness of 7 mm was associated with a fibrinogen level of approximately 2 g/l [10]. During massive blood loss replacement, fibrinogen may be the first coagulation factor to decrease critically [280]. During postpartum haemorrhage, fibrinogen plasma concentration is the only coagulation parameter independently associated with progress toward severe bleeding, with a level less than 2 g/l having a positive predictive value of 100% [281]. Blood loss and blood transfusion needs were also found to inversely correlate with preoperative fibrinogen levels in coronary artery bypass graft surgery [282].

During serious perioperative bleeding, fibrinogen treatment (2 g, range 1 to 5 g) was associated with a reduction in allogeneic blood product transfusion [283]. The fibrinogen concentration before treatment was 1.4 g/l (IQR 1.0 to 1.8 g/l) rising to 2.4 g/l (IQR 2.1 to 2.6 g/l) after fibrinogen substitution [283]. An observational study suggests that fibrinogen substitution can improve survival in combat-related trauma [284]. An RCT in patients undergoing radical cystectomy with excessive blood loss has shown that postoperative blood transfusions could be reduced by the administration of 45 mg/kg fibrinogen at a mean pre-treatment fibrinogen level of 1.7 ± 0.3 g/l rising to 2.4 ± 0.1 g/l following fibrinogen substitution [285].

Fibrinogen administration using thrombelastometry as guidance may be preferable to measuring fibrinogen levels in the laboratory. Some methodological issues in the various laboratory methods to measure fibrinogen concentration remain [286,287], and in the presence of artificial colloids such as hydroxyethyl starch, even the most frequently recommended method [287], the Clauss method, significantly overestimates the actual fibrinogen concentration [288].

It is not known whether the administration of fibrinogen via factor concentrate, cryoprecipitate or FFP is associated with a post-traumatic venous thrombotic risk. However, fibrinogen levels are expected to rise to a level of approximately 7 g/l after major surgery and trauma [289,290] even without intra-operative fibrinogen administration, and the effect of intra-operative fibrinogen administration on postoperative fibrinogen levels is unknown at the present time. Interestingly, intra-operative administration of 45 mg/kg fibrinogen concentrate in patients undergoing cystectomy resulted in higher early postoperative fibrinogen levels but already at 24 hours post-operation fibrinogen levels were identical in patients with and without intra-operative fibrinogen administration [285]. Similarly, 24 hour fibrinogen levels were identical in patients who received and those who did not receive 2 g of fibrinogen prior to coronary artery bypass graft surgery [291]. This result is in keeping with the study by Weinkove and Rangarajan, who found no thrombotic risk in patients treated with fibrinogen concentrate due to acquired hypofibrinogenemia (fibrinogen <1.5 g/l) [292].

#### Pharmacological agents

An increasingly large body of evidence supports the use of antifibrinolytic agents for the management of bleeding in elective surgery and cardiac surgery patients. For the purpose of these guidelines, we have assumed that these effects are transferable to trauma patients, and our recommendations are based upon this unproven assumption. Since the last guidelines were written, aprotinin has been associated with patient safety issues, with an increased rate of renal disease and mortality when compared with the lysine analogues in a large clinical trial and is therefore no longer recommended [293-296].

#### Antifibrinolytic agents

##### Recommendation 27

We suggest that antifibrinolytic agents be considered in the bleeding trauma patient (Grade 2C). We recommend monitoring of fibrinolysis in all patients and administration of antifibrinolytic agents in patients with established hyperfibrinolysis (Grade 1B). Suggested dosages are tranexamic acid 10 to 15 mg/kg followed by an infusion of 1 to 5 mg/kg per hour or ε-aminocaproic acid 100 to 150 mg/kg followed by 15 mg/kg/h. Antifibrinolytic therapy should be guided by thrombelastometric monitoring if possible and stopped once bleeding has been adequately controlled (Grade 2C).

##### Rationale

Tranexamic acid (trans-4-aminomethylcyclohexane-1-carboxylic acid) is a synthetic lysine analogue that is a competitive inhibitor of plasmin and plasminogen. Tranexamic acid is distributed throughout all tissues and the plasma half-life is 120 minutes. There is large variation in the dose employed. *In vitro *studies have suggested that a dose of 10 μg/ml is required to inhibit fibrinolysis [297]. Studies of plasma levels [298] confirmed that the Horrow regimen (10 mg/kg followed by 1 mg/kg per hour) [299], shown to reduce blood loss in cardiac surgery, attained these levels. Other studies have used boluses of up to 5 g per patient with no ill effect [300].

ε-aminocaproic acid is also a synthetic lysine analogue that has a potency 10-fold weaker than that of tranexamic acid. It is therefore administered in a loading dose of 150 mg/kg followed by a continuous infusion of 15 mg/kg/h. The initial elimination half-life is 60 to 75 minutes and it must therefore be administered by continuous infusion in order to maintain therapeutic drug levels until the bleeding risk has diminished.

The clear efficacy of antifibrinolytic agents in reducing bleeding in elective surgery and especially in cardiac surgery has been shown in numerous clinical trials [301-305]. The benefits of antifibrinolytics in these situations where hyperfibrinolysis is not usually seen, suggests that under normal circumstances when a patient has a bleeding vessel, there is low-grade fibrinolytic turnover that exacerbates bleeding. Thus, fibrinolysis is 'switched off' and less bleeding results. It may be possible to extrapolate the benefits of antifibrinolytic agents to bleeding secondary to trauma, although this assumption is not backed by any published data that suggest that the haemostatic response to trauma is similar to the haemostatic response to elective surgery. There is insufficient evidence from RCTs of antifibrinolytic agents in trauma patients to either support or refute a clinically important treatment effect. The efficacy of tranexamic acid in trauma has been assessed by the Clinical Randomisation of an Antifibrinolytic in Significant Haemorrhage (CRASH) II study, in which 20,000 trauma patients worldwide were randomly assigned to 1 g of tranexamic acid for a period of 10 minutes followed by 1 g infused for a period of eight hours. This results of this trial are due to be published in 2010 [306].

The risk of precipitated thrombosis with the use of the lysine analogues tranexamic acid and ε-aminocaproic acid has been of major theoretical concern; however, the Cochrane review of antifibrinolytics cites studies that included more than 8,000 patients receiving lysine analogues and demonstrated no increased risk of either arterial or venous thrombotic events [307]. The lysine analogues are renally excreted and accumulate in individuals with renal failure, therefore dosage should be reduced in patients with renal failure. In practice, mild degrees of renal failure do not seem to affect outcome.

#### Activated recombinant coagulation factor VII

##### Recommendation 28

We suggest that the use of recombinant activated coagulation factor VII (rFVIIa) be considered if major bleeding in blunt trauma persists despite standard attempts to control bleeding and best-practice use of blood components (Grade 2C).

##### Rationale

rFVIIa is not a first-line treatment for bleeding and will be effective only once sources of major bleeding have been controlled. Once major bleeding from damaged vessels has been stopped, rFVIIa may be helpful to induce coagulation in areas of diffuse small vessel coagulopathic bleeding. rFVIIa should be considered only if first-line treatment with a combination of surgical approaches, best-practice use of blood products (RBCs, platelets, FFP and cryoprecipitate/fibrinogen resulting in Hct above 24%, platelets above 50,000 × 10^9^/l and fibrinogen above 1.5 to 2.0 g/l), the use of antifibrinolytics and correction of severe acidosis, severe hypothermia and hypocalcaemia fail to control bleeding. Because rFVIIa acts on the patient's own coagulation system, adequate numbers of platelets and fibrinogen levels are needed to allow a thrombin burst to be induced by the pharmacological, supraphysiological doses of rFVIIa through direct binding to activated platelets [308,309]. pH and body temperature should be restored as near to physiological levels as possible because even small reductions in pH and temperature result in slower coagulation enzyme kinetics [206,207,310]. Moreover, hypocalcaemia is frequently present in severely injured patients [251] and so monitoring of ionised calcium is necessary and administration of intravenous calcium may be required [311].

Despite numerous case studies and series reporting that treatment with rFVIIa can be beneficial in the treatment of bleeding following trauma, there are few high-quality studies [312-315]. A multi-centre, randomised, double-blind, placebo-controlled study examined the efficacy of rFVIIa in patients with blunt or penetrating trauma [316] and showed that patients with blunt trauma who survived for more than 48 hours, assigned to receive rFVIIa 200 μg/kg, after they had received eight units of RBCs, and a second and third dose of 100 μg/mg one and three hours later; had a reduction in RBC transfusion requirements and the need for massive transfusions (>20 units of RBCs), compared with placebo. They also had a significantly reduced incidence of ARDS. In contrast, there were no significant effects in the penetrating trauma patients in this study, although trends toward reduced RBC requirements and fewer massive transfusions were observed.

The required dose(s) of rFVIIa is still under debate. Whereas the above dosing recommendation is based on the only published RCT available in trauma patients and is also recommended by a group of European experts [317], Israeli guidelines based on findings from a case series of 36 patients who received rFVIIa on a compassionate-use basis in Israel [313] propose an initial dose of 120 μg/kg (between 100 and 140 μg/kg) and (if required) a second and third dose. Pharmacokinetic modelling techniques have shown that the dose regimen for rFVIIa treatment used in the above cited RCT is capable of providing adequate plasma levels of drug to support haemostasis [318].

If rFVIIa is administered, the patient's next of kin should be informed that rFVIIa is being used outside the currently approved indications (off-label use), especially because the use of rFVIIa may increase the risk of thromboembolic complications [319]. Recent data from a meta-analysis performed by the manufacturer on pooled data from placebo-controlled trials outside current approved indications in various clinical settings included over 2,000 patients and showed a higher risk of arterial thromboembolic adverse events (5.6% in patients receiving rFVIIa versus 3.0% in placebo-treated patients) [320].

#### Prothrombin complex concentrate

##### Recommendation 29

We recommend the use of prothrombin complex concentrate for the emergency reversal of vitamin K-dependent oral anticoagulants (Grade 1B).

##### Rationale

Despite the increasing off-license use of PCC, there are no studies to support its use other than in haemophilia [321-323] or for the rapid reversal of the effect of oral vitamin K antagonsists [324-326]. With an ageing population, more trauma patients are likely to be taking vitamin K antagonists, therefore every trauma unit should have an established management policy for these patients. The comparison between outcomes other than speed of reversal of anticoagulation between FFP and PCC has not been established; several clinical trials are in progress, although none relates specifically to trauma patients. Despite some clinical recommendations [327], no clinical studies have been performed to determine whether administration of PCC is efficacious and safe in managing bleeding in trauma patients who are not on vitamin K antagonists, although a swine model suggests that there may be some advantages [328].

Because the use of PCC carries the theoretical increased risks of both venous and arterial thrombosis during the recovery period [329,330], the use of thromboprophylaxis is recommended in patients who have received PCC. Because there are variations in the production of PCC, the dosage should be determined according to the instructions of the individual manufacturer [331]. Research is urgently required to assess whether PCC has a place in the management of the bleeding trauma patient.

#### Desmopressin

##### Recommendation 30

We do not suggest that desmopressin be used routinely in the bleeding trauma patient (Grade 2C). We suggest that desmopressin be considered in refractory microvascular bleeding if the patient has been treated with platelet-inhibiting drugs such as acetylsalicylsalicylic acid (Grade 2C).

##### Rationale

Desmopressin (1-deamino-8-D-arginine) enhances platelet adherence and platelet aggregate growth on human artery subendothelium and was originally licensed for use in von Willebrand disease [332], a disease that occurs in roughly 1 in 100 patients and in whom desmopressin is routinely used. In 1986 the first study was published stating that desmopressin reduces blood loss after cardiac surgery by 30% in comparison with placebo [333]; however, subsequent studies showed controversial results. Two recently published meta-analyses [334,335] were able to demonstrate either a trend towards a reduced blood loss [334] or a small significant reduction in blood transfusion requirements (-0.29 (-0.52 to -0.06) units per patient), but neither study could demonstrate any effect on the course of the disease or mortality. At the same time, concerns arose with respect to possible thromboembolic complications of this procoagulant drug. Whereas Ozal and colleagues described a 2.4-fold increase in risk for myocardial infarction with desmopressin [336], the last meta-analysis from 2008 could not identify a significant increase in myocardial infarction or thrombosis associated with desmopressin. Both meta-analyses stress the need for more RCTs to allow for clear recommendations. On the other hand, patients may benefit from desmopressin if they have been pre-treated with platelet-inhibiting drugs, for example acetylsalicylsalicylic acid [337].

No studies have investigated the effect of desmopressin in the trauma patient, and there is great uncertainty as to whether the results of studies involving non-trauma patients can be applied to bleeding following trauma. Therefore, only a weak recommendation can be made.

#### Antithrombin III

##### Recommendation 31

We do not recommend the use of antithrombin concentrates in the treatment of the bleeding trauma patient (Grade 1C).

##### Rationale

Antithrombin concentrates are indicated in inherited and acquired antithrombin deficiency. Although antithrombin deficiency does occur in consumptive coagulopathy, this is not an isolated condition; all coagulation factors and physiological anticoagulants undergo consumption under these circumstances. The best replacement therapy is FFP. Clinical studies of antithrombin concentrate in severe blunt trauma and in critical care have shown no benefit [338,339].

## Discussion

This guideline for the management of the bleeding trauma patient is based on a critical appraisal of the published literature, a re-appraisal of the recommendations we published three years ago and a consideration of current clinical practice in areas in which RCTs will never be performed for practical or ethical reasons. In the process of generating this updated version of the guideline, we identified a number of scientific questions that have emerged or were not addressed previously and have developed recommendations to cover these issues. The new and revised recommendations included here reflect both newly available evidence and shifts in general clinical practice. As bedside testing, particularly thrombelastogram-based methodology, and multi-slice CT have become more established in the emergency department setting, we felt a need to update our guideline to discuss the use of these new technologies. We also include new recommendations on the use of tourniquets as an adjunct to halt life-threatening open extremity injuries, ionised calcium monitoring and treatment, and the use of local haemostatic agents and desmopressin in the bleeding trauma patient. Our recommendations have also been updated to reflect the recent removal of aprotinin as an antifibrinolytic agent from the market. The final draft of this document omitted a draft recommendation on the use of coagulation factor XIII because, although the author group feels that this agent may play a role in the haemostatic management of trauma patients in future, the present lack of evidence in trauma and means of monitoring the therapeutic effect of this compound in many hospitals precludes a specific recommendation at this time.

Although the level of scientific evidence has improved in some areas, particulary those that have come under closer scruitiny in the context of ongoing military conflicts, other areas remain devoid of high-level evidence. Although evidence gathered in a military setting may or may not be readily transferable to the civilian setting, recent experience has shown that there is a need for uniform practices in the management of the traumatically injured patient [340]. This observation renders the need for best-practice guidelines even more acute.

We have excluded animal studies from the evidence considered here, and maintain our opinion that humans are the best subjects in whom to study human post-traumatic injury [341]. We also continue to concur that in the absence of evidence to the contrary, children and elderly adults, with the exception of those who have been treated with anticoagulant or antiplatelet agents, should generally be managed in the same manner as the normal adult patient. Given the risk of thrombembolic complications, we suggest that the application of pro-coagulant measures be ceased once haemostasis has been achieved.

**Figure 1:**
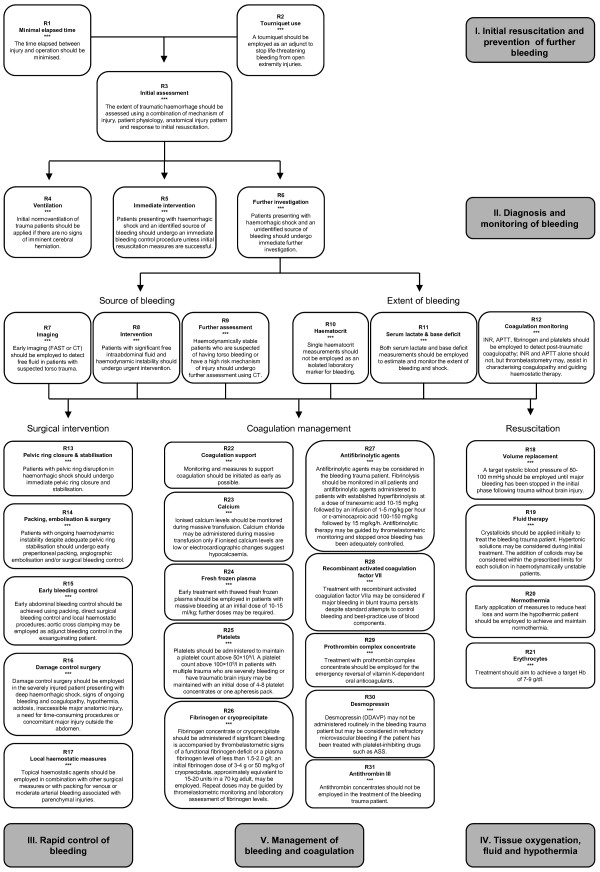
**Flow chart of treatment modalities for the bleeding trauma patient discussed in this guideline**. APTT: activated partial thromboplastin time; ASS: acetylsalicylsalicylic acid; CT: computed tomography; FAST: focused abdominal sonography for trauma; Hb: haemoglobin; INR: international normalised ratio.

All of the recommendations presented here were formulated according to a consensus reached by the author group and the professional societies involved. Figure 1 graphically summarises the recommendations included in this guideline. We have employed the GRADE [14-16] hierarchy or evidence to formulate each recommendation because it allows strong recommendations to be supported by weak clinical evidence in areas in which the ideal clinical RCTs may never be performed. To minimise the bias introduced by individual experts, we employed a nominal group process to develop each recommendation and several Delphi rounds to reach an agreement on the questions to be considered and to reach a final consensus on each recommendation. To ensure that the process included input from all of the relevant specialties, the group comprised a multidisciplinary pan-European group of experts, including the active involvement of representatives from five of the most relevant European professional societies.

## Conclusions

A multidisciplinary approach to management of the traumatically injured patient remains the cornerstone of optimal patient care, and we have made an effort to formulate this guideline in a manner that is widely applicable to a variety of settings in clinical practice. As the volume and level of evidence in this field accumulates, the current state-of-the-art as reflected in this guideline will need to evolve accordingly.

## Key messages

• This clinical practice guideline provides evidence-based recommendations developed by a multidisciplinary task force with respect to the acute management of the bleeding trauma patient, which when implemented may improve patient outcomes.

• Coagulation monitoring and measures to support coagulation should be implemented as early as possible following traumatic injury and used to guide haemostatic therapy.

• A damage control approach to surgical procedures should guide patient management, including closure and stabilisation of pelvic ring disruptions, packing, embolisation and local haemostatic measures.

• This guideline reviews appropriate physiological targets and suggested use and dosing of fluids, blood products and pharmacological agents in the bleeding trauma patient.

• A multidisciplinary approach to management of the traumatically injured patient remains the cornerstone of optimal patient care.

## Abbreviations

ACS: abdominal compartment syndrome; APTT: activated partial thromboplastin time; ARDS: acute respiratory distress syndrome; ATLS: Advanced Trauma Life Support; CT: computed tomography; DPL: diagnostic peritoneal lavage; FAST: focused abdominal sonography for trauma; FFP: fresh frozen plasma; GCS: Glasgow coma score; GRADE: Grading of Recommendations Assessment: Development and Evaluation; Hb: haemoglobin; Hct: haematocrit; INR: international normalised ratio; IQR: interquartile range; MeSH: medical subject heading; MSCT: multi-slice computed tomography; NIH: National Institutes of Health; PCC: prothrombin complex concentrate; PEEP: positive end-expiratory pressure; PT: prothrombin time; RBC: red blood cell; RCT: randomised controlled trial; rFVIIa: recombinant activated coagulation factor VII; TBI: traumatic brain injury.

## Competing interests

RR has received honoraria for consulting or lecturing from CSL Behring, Novo Nordisk, Bayer, Air Liquide and Eli Lilly and has received research grant funding from AGA-Linde, Air Liquide, Novo Nordisk, Eli Lilly and Glaxo Wellcome. BB has received honoraria for consulting or lecturing from Novo Nordisk, CSL Behring and Sangart. VC has received honoraria for consulting or lecturing from Fresenius (Czech Republic), Schering-Plough (Czech Republic), B. Braun (Czech Republic) and Novo Nordisk (Czech Republic). TJC has received research funding from the UK National Institute for Health Research, BOC Linde and the Mid Anglian GP Accident Service. He is a trustee of BRAKE (a road safety charity) and the College of Emergency Medicine. JD has received honoraria for consulting or lecturing from Novo Nordisk, LFB Biomédicaments and Hutchinson Technology. EF-M has has received honoraria for consulting or lecturing from Sangart and PULSION Medical Systems. BJH has received honoraria for consulting or lecturing from Bayer, Boehringer Ingelheim, Sanofi Aventis and Novo Nordisk. RK has no competing interests to declare. GN has received honoraria for consulting or lecturing from Novo Nordisk and Sangart and institutional research grant funding from Novo Nordisk. EN has received honoraria for consulting or lecturing from Biotest (Dreieich, Germany), Javelin Pharma (NY, USA), Novo Nordisk (Denmark), MSD Sharp & Dohme (Haar), Pfizer (Berlin), AstraZeneca (Wedel), B. Braun (Melsungen) and Bristol Myers Squibb (Munich, Germany) and has received institutional support from Mundipharma (Limburg), Cook Ltd. (Bloomington, IN, USA), QRX Pharma (Bedminster, NJ, USA), Ethicon (Norderstedt), KCI (Amstelveen, NL) and Sanofi (Berlin). YO has received institutional support from LFB (Laboratoire français du Fractionnement et des Biotechnologies), Octapharma and Novo Nordisk. LR been involved in educational courses on bleeding control supported by Baxter. AS has no competing interests to declare. PFS has received honoraria for consulting or lecturing from Synthes, Stryker Spine and Novo Nordisk. JLV has received honoraria for consulting or lecturing from AstraZeneca, Edwards Lifesciences, Pfizer, Astellas, Eli Lilly, Ferring, GSK, the Medicines group amd Novo Nordisk and has received research grant funding from AM Pharma, Artisan, Astellas, Curacyte, Eli Lilly, Esai and Novo Nordisk. DRS has received honoraria or travel support for consulting or lecturing from Abbott AG (Baar, Switzerland) Alliance Pharmaceutical Corp. (San Diego, CA, USA) AstraZeneca AG (Zug, Switzerland) Bayer (Schweiz) AG (Zürich, Switzerland) B. Braun Melsungen AG (Melsungen, Germany), Boehringer Ingelheim (Schweiz) GmbH (Basel, Switzerland), CSL Behring GmbH (Hattersheim am Main, Germany), Curacyte AG (Munich, Germany) Fresenius SE (Bad Homburg v.d.H., Germany), Galenica AG ((including Vifor SA, Villars-sur-Glâne) Bern, Switzerland), GlaxoSmithKline GmbH & Co. KG (Hamburg, Germany), Janssen-Cilag AG (Baar, Switzerland), Novo Nordisk A/S (Bagsvärd, Denmark), Octapharma AG (Lachen, Switzerland), Organon AG (Pfäffikon/SZ, Switzerland), Oxygen Biotherapeutics (Costa Mesa, CA, USA), Pentapharm GmbH (Munich, Germany), Roche Pharma (Schweiz) AG (Reinach, Switzerland) and Schering-Plough International, Inc. (Kenilworth, NJ, USA). His academic department currently receives grant support from the University of Zurich, the Research Award Center for Zurich Integrative Human Physiology, the Swiss National Science Foundation, the Swiss Foundation for Anesthesia Research, the European Society of Anaesthesiology (ESA), the Swiss Society of Anesthesiology and Reanimation (SGAR), the Gebert Ruef Foundation, the Swiss Life Foundation, the Olga Mayenfisch Foundation, Abbott AG Switzerland, B. Braun Switzerland, UBS Switzerland, Stiftung für Staublungenforschung, Switzerland.

The ABC-T European medical education initiative is managed by Physicians World Europe GmbH (Mannheim, Germany) and supported by educational grants from Novo Nordisk.

## Authors' contributions

All of the authors participated in the formulation of questions to be addressed in the guideline, screening of abstracts and literature, face-to-face and remote consensus-finding processes, drafting, review, revision and approval of the manuscript.

## Authors' information

RR serves as chair of the Advanced Bleeding Care in Trauma (ABC-T) European medical education initiative. VC is a member of the ABC-T European medical education initiative faculty. TJC is a member of the ABC-T European medical education initiative faculty. JD is a member of the ABC-T European medical education initiative faculty. EF-M is a member of the ABC-T European medical education initiative faculty. PFS is a member of the ABC-T European medical education initiative faculty. RK represented the European Society of Trauma and Emergency Surgery (ESTES) on the ABC-T Task Force. YO represented the European Society of Intensive Care Medicine (ESICM) on the ABC-T Task Force. LR represented the European Society for Emergency Medicine (EuSEM) on the ABC-T Task Force. AS represented the European Shock Society (ESS) on the ABC-T Task Force. DRS serves as co-chair of the Advanced Bleeding Care in Trauma (ABC-T) European medical education initiative and represented the European Society of Anaesthesiology (ESA) on the ABC-T Task Force.

## Supplementary Material

Additional file 1 **MeSH terms and limits applied to address guideline literature queries - 2009**. Word file containing MeSH terms and limits applied to address guideline literature queries. Click here for file
